# Bilocated Intracochlear Electrocochleography During Cochlear Implantation to Provide Surgical Feedback

**DOI:** 10.1097/MAO.0000000000004675

**Published:** 2025-10-16

**Authors:** Leanne Sijgers, Rahel Bertschinger, Marlies Geys, Ahmet Kunut, Christof Röösli, Alexander Huber, Flurin Pfiffner, Adrian Dalbert

**Affiliations:** Department of Otorhinolaryngology, Head and Neck Surgery, University Hospital Zurich, University of Zurich, Zurich, Switzerland

**Keywords:** Bioelectricity, Biosignals, Cochlear implant, Electrophysiology, intraoperative monitoring

## Abstract

**Introduction:**

Recent advancements toward minimizing surgical trauma and preserving residual hearing during cochlear implantation have promoted the use of intracochlear electrocochleography (ECochG) for intraoperative surveillance. However, variations in the distribution of response generators throughout the cochlea complicate the interpretation of signal changes during electrode array insertion. This exploratory study aimed to investigate whether simultaneous recordings from 2 locations within the cochlea could address this issue. This was done by (1) comparing recordings acquired simultaneously from an apical and a more basal electrode contact during atraumatic electrode array insertions; and (2) comparing these response patterns with preoperative pure-tone audiograms.

**Materials and methods:**

In 10 standard CI recipients, ECochG recordings were obtained during stepwise insertion of a short-temporary electrode array. Simultaneous intracochlear recordings were acquired from 2 contacts separated by 4.2 mm in response to 500 Hz tone bursts. For both electrode contacts, the differences between responses to alternating-polarity stimuli, hereafter named “ECochG responses,” were derived. After completion of the recordings, the temporary electrode array was removed and a standard CI electrode array inserted.

**Results:**

In 6 of 10 participants, ECochG response amplitude decreases of ≥3 dB were recorded at the more basal electrode contact. In 4 cases, these amplitude drops were preceded by amplitude drops recorded at the apical electrode contact, with both occurring no more than 1.7 mm apart along the cochlear duct. Trauma would be expected to produce a simultaneous amplitude drop—at different recording locations—for both electrodes. In contrast, a drop at the basal electrode that is preceded by a drop in the ECochG recording at the apical electrode, with both drops occurring at approximately the same location, is likely atraumatic. These atraumatic drops may also be associated with large phase shifts. Overall, the ECochG response tracks recorded at the apical and more basal contact were similar, while the ECochG response patterns and audiogram did not show a strong resemblance.

**Conclusion:**

The proposed approach could facilitate the detection of ECochG response changes relevant for predicting hearing preservation during cochlear implantation. By distinguishing between simultaneous and sequential amplitude drops, this method could provide additional insights into the atraumatic nature of certain ECochG response changes.

## Introduction

Growing interest in minimizing surgical trauma and preserving residual hearing during cochlear implantation has led to the investigation of intracochlear electrocochleography (ECochG) for intraoperative surveillance. Real-time ECochG measurements through the implant’s electrodes have been facilitated by cochlear implant (CI) manufacturers using back-telemetry.^1–3^ During CI insertion, alternating-polarity acoustic stimuli are typically used to collect responses from the array’s most apical electrode. Currently, feedback is provided based on a detected reduction in the amplitude of the alternating-polarity stimuli responses’ difference, hereafter named “ECochG response.”^4,5^ This component of ECochG primarily signifies the cochlear microphonic response from hair cells and has been identified as sensitive to cochlear trauma.^6^

However, ECochG responses can change due to many factors. When using low-frequency tone bursts for acoustic stimulation, ECochG response amplitude decreases unrelated to cochlear trauma often occur near full insertion. This is potentially due to destructive interferences between hair cells from the stimulation frequency’s resonance location and more basal hair cells, which are known to move out of phase.^7,8^ Variations in response generator distribution throughout the cochlea could also result in atraumatic ECochG amplitude decreases. Finally, fixation of the basilar membrane by the electrode array could disrupt the traveling wave and lead to a loss in ECochG response,^9,10^ which may potentially be resolved at later insertion stages.

Recent efforts to enhance the predictive power of ECochG have concentrated on using multifrequency stimuli,^11,12^ using simultaneous intracochlear and extracochlear recordings,^13,14^ adjusting the intracochlear recording electrode in real time,^3^ and including additional ECochG components such as the response’s phase for analysis.^7,15,16^ The rationale behind these methods is that they provide additional information to determine whether an ECochG amplitude decrease reflects trauma or not. It was shown that simultaneous decreases in ECochG responses to stimuli with different frequencies,^11,12,17^ or in intracochlear and extracochlear ECochG recordings,^13,14^ may better reflect cochlear trauma than standard intracochlear ECochG recordings using 500 Hz tone bursts. Study results also suggest that intracochlear ECochG amplitude decreases more likely reflect trauma in the absence of large phase changes or harmonic distortions,^7,14^ and when they occur in the later stages of electrode array insertion.^14,18^

The present study aimed to investigate whether simultaneous intracochlear ECochG recordings from 2 locations could help distinguish traumatic and atraumatic response amplitude drops. Intracochlear ECochG responses were acquired using a 2-channel external-evoked potential (EP) recording device connected to a short, multicontact, temporary electrode array. The decision to use this recording setup instead of CI recording telemetry in this exploratory study was driven by the advantages of a more sensitive amplifier and greater flexibility in recording parameters offered by an external EP device, such as the possibility to select 2 electrodes for obtaining simultaneous recordings. In a previous study, in which we recorded ECochG responses during insertion and retraction of the same electrode array in 10 participants, we could show that the insertions with this array were atraumatic in all cases as the response changes recorded during array insertion were reversible.^13^ In the present study, we (1) compared recordings acquired simultaneously from an apical and a more basal electrode contact during atraumatic electrode array insertions, and (2) compared response patterns with preoperative pure tone audiograms. Since postoperative residual hearing loss is expected to result from either the insertion of the CI electrode array—following the retraction of the temporary electrode—or from postoperative processes, no systematic comparisons were made between ECochG recordings and postoperative audiograms. We hypothesized that the ECochG response of a more basal contact should follow the response pattern of an apical contact with some delay if no changes of inner ear function occur during insertion of the array.

## Materials and methods

Seventeen individuals with some degree of residual hearing at 500 Hz undergoing CI surgery were prospectively enrolled in this exploratory study. Seven participants were subsequently excluded because no ECochG responses could be detected in their intraoperative recordings. The remaining participants are referred to as participant P01 up to P10. Following the temporary insertion of the Auditory Nerve Test System (ANTS) electrode array (MED-EL, Innsbruck, Austria), all 10 participants received CIs with free-fitting lateral wall arrays, with P01 receiving a FLEX24 electrode array and all other participants receiving a FLEX28 array (MED-EL). The study was approved by the local Ethical Committee (KEK-ZH-2020-01025) and conducted in concordance with the declaration of Helsinki. All participants provided written informed consent before surgery.

Preoperative pure tone audiometry was conducted during a routine clinical appointment. The time between the audiometric assessment and surgery was around 1 week for P01, P02, P04, and P05, 3 months for P03, 6 months for P07, P08, P09, P10, and 1 year for P06. The air conduction threshold values were determined at 0.25, 0.5, 1, 2, 3, 4, 6, and 8 kHz, in accordance with ISO 8253-1:2010. To calculate the pure tone average (PTA) from these frequencies, the maximum output of the audiometer plus 5 decibels (dB) was used as a threshold value if no response was present at the maximum output of the audiometer [90 dB hearing level (HL) for 0.25 and 8 kHz, 110 dB HL for 6 kHz, and 115 dB HL for 0.5, 1, 2, 3, and 4 kHz].

### Measurement protocol

After induction of anesthesia, an insert earphone (Biologic Systems, Mundelein, IL) and a probe microphone (ER-7C; Etymotic Inc., Elk Grove Village, IL) were placed in the ipsilateral ear canal for acoustic stimulation and sound pressure verification. Following a standard transmastoid facial recess approach, the ANTS electrode array was temporarily inserted through the round window in 8 to 12 steps, with one step measuring ∼1.7 mm. ECochG responses were recorded from 2 intracochlear electrode contacts at each step using the Navigator Pro stimulation and recording device (Biologic Systems) while holding the electrode array in place. Following the recordings at full insertion, a noise floor recording was made at both electrode contacts by disconnecting the insert earphone from the loudspeaker and repeating the ECochG measurement. After completion of the recordings, the temporary electrode array was removed and a standard CI inserted.

### Measurement specifics

**Figure 1:**
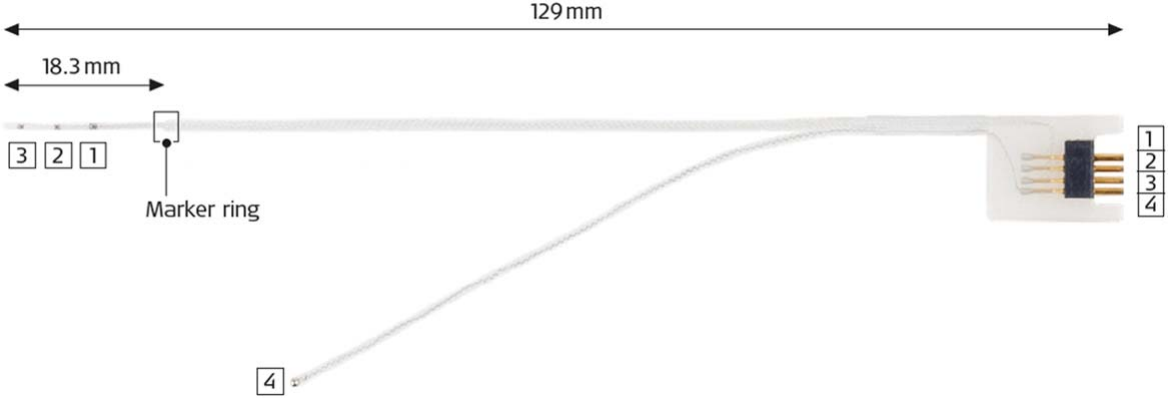
The Auditory Nerve Test System electrode array used to acquire intracochlear ECochG responses. The array has three single contacts spaced 4.2 mm apart, of which the 2 most apical ones, referred to as “apical” and “basal” electrode contact in this manuscript, are used to obtain the recordings.

Figure 1 shows the ANTS electrode array that was used to perform ECochG recordings. The array’s 2 most apical electrodes, spaced 4.2 mm apart, were used to obtain simultaneous recordings from 2 intracochlear locations. Needle electrodes placed in the contralateral preauricular region and on the forehead were used as inverting and ground electrodes, respectively, to conduct ECochG recordings using a horizontal montage. A connector cable and custom-made adapter provided by MED-EL were used to connect the apical and middle electrode contact of the ANTS electrode array, hereafter referred to as “apical” and “basal” electrode contact, to the 2 positive recording channels of the Navigator Pro device. To minimize electrical artifacts resulting from the antenna effect of the floating unused ANTS electrode contacts, the reference contact was positioned beneath the temporalis muscle, and a short-circuit was established between the basal electrode contact and the reference electrode. At the first few insertion steps, where only the apical electrode contact was inside the cochlea, the basal electrode was grounded through an adhesive electrode placed on the participant’s forehead.

At each step, acoustic stimulation was performed using 800 tone bursts with alternating polarity, a frequency of 500 Hz and an intensity of 112 to 125 dB SPL. The sound pressure level was chosen based on preoperative residual hearing and the size of the ECochG response recorded at the first step and was kept constant throughout all recordings in 1 participant. The probe microphone connected to an oscilloscope was used to verify the sound pressure close to the tympanic membrane in real-time. The stimuli had a plateau phase of 10 cycles and an additional 2-cycle rise and fall time shaped by a Blackman window, leading to a total stimulus duration of 28 ms. The responses to alternate-polarity stimuli were separately averaged and stored.

### Data analysis

The recordings were analyzed using MATLAB R2022b (MathWorks Inc., Natick, MA) and visualized using R. The ECochG responses were derived by subtracting the averaged responses to alternating-polarity stimuli. A Fast Fourier Transform (FFT) was performed on the ECochG response. The amplitude and phase of each ECochG response were obtained from the FFT bin at 500 Hz, hereafter referred to as “ECochG amplitude” and “ECochG phase.” A measurement was considered valid if the FFT amplitude of the ECochG response was at least 6 dB above the amplitude of the corresponding FFT bin in the noise floor recording, as in a previous study.^14^ Participants were included if they had valid ECochG recordings at a minimum of 2 steps for both apical and basal electrode recordings.

To compare the recordings made using the apical and basal electrode contact and their respective intracochlear locations, insertion depths of 17.8 and 13.6 mm were assumed for the apical and basal electrode contact at full insertion, based on the manufacturer’s specifications. The insertion depth for the first recording with each electrode was assumed to be 0.8 mm. Linear interpolation was then used to estimate the insertion depth of each electrode contact at the different insertion steps. For visualization purposes, the insertion angle and tonotopic region for all recording electrode locations were derived from the insertion depths and postoperative imaging using OTOPLAN version 3 (MED-EL). It was assumed that the trajectories of the ANTS and CI electrode array would mimic each other, so that the relationships between insertion depth, insertion angle and tonotopic region of the ANTS recording electrodes could be derived from the final CI electrode placement. The ECochG amplitudes were then plotted against insertion angle and tonotopic frequency, together with the participants’ preoperative audiograms, using the ZH-ECochG Bode Plot.^19^ For participants P02 and P09, the insertion angle and tonotopic frequency were derived by averaging the parameters of the other eight participants because postoperative imaging of sufficient quality was not available.

The participants’ ECochG responses were categorized into groups based on whether the basal electrode recorded no drop, an expected drop, or an unexpected drop. A drop is defined as a change of at least 3 dB in the ECochG response amplitude with respect to a previous maximum.^14^ A drop is expected at the basal electrode when it is preceded by a drop in the ECochG recording at the apical electrode, and the locations of both drops are within 1.7 mm of each other (the approximate insertion step size). Patterns in apical ECochG response amplitudes were compared with the preoperative audiogram using visual inspection.

## Results

Table 1 shows the demographic and clinical variables of study participants P01 up to P10, as well as the preoperative PTA and selected and detected sound pressure levels of the acoustic stimuli used for obtaining ECochG responses. Differences between selected and detected acoustic stimulation levels can result from variations in outer ear acoustics or earfoam placement within the ear canal.

**Table 1:** Participant demographics, side of implantation, hearing loss etiology, preoperative pure tone average (PTA), selected acoustic stimulation level used for obtaining ECochG responses, and sound pressure level detected using the probe microphone

Participant	Age	Sex	Side	Etiology	Preoperative PTA	Selected acoustic stimulation level (dB SPL)	Detected sound pressure level (dB SPL)
P01	73	Female	Left	Idiopathic	91.25	115	Technical recording issues
P02	74	Male	Right	Meniere disease	69.38	120	124
P03	61	Male	Right	Sudden sensorineural hearing loss	96.25	125	118
P04	60	Female	Left	Idiopathic	76.88	120	122
P05	69	Female	Left	Idiopathic	90.63	120	121
P06	55	Female	Left	Idiopathic	76.25	120	122
P07	76	Male	Right	Idiopathic	82.50	125	126
P08	71	Female	Right	Idiopathic	84.38	112	128
P09	76	Male	Right	Idiopathic	72.50	120	122
P10	82	Male	Left	Stable vestibular Schwannoma	98.13	120	124

The PTA was calculated from the following frequencies: 0.25, 0.5, 1, 2, 3, 4, 6, and 8 kHz. dB stands for decibel, and SPL stands for sound pressure level. For participant P10, the Schwannoma itself was not treated since it had been stable in size for many years.

**Figure 2:**
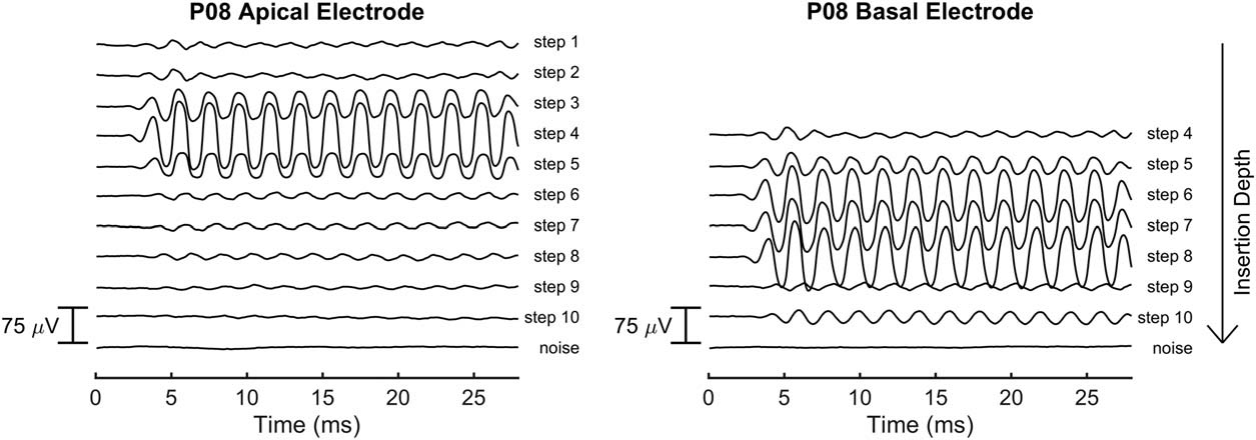
Intracochlear ECochG responses for participant P08 recorded at the different insertion steps using the array’s apical and basal electrode. The basal electrode contact entered the cochlea at step 4.

Figure 2 shows the exemplary ECochG responses of P08 recorded using the apical and basal electrode contacts. In this study participant, the temporary electrode array was inserted in ten steps, with the basal electrode contact entering the cochlea at step 4. An amplitude drop at the basal electrode contact was detected at step 9, while the apical electrode recorded drops at steps 5 and 6, and again at steps 9 and 10. The approximated insertion depths were 11.47 mm when the basal electrode recorded a drop at step 9, and 10.24 mm for the corresponding amplitude drop at the apical electrode at step 6. As the amplitude drop at the more basal electrode contact occurred within 1.7 mm of a detected amplitude drop at the apical electrode contact, this participant’s recordings were classified into the “expected amplitude drop” group.

**Figure 3:**
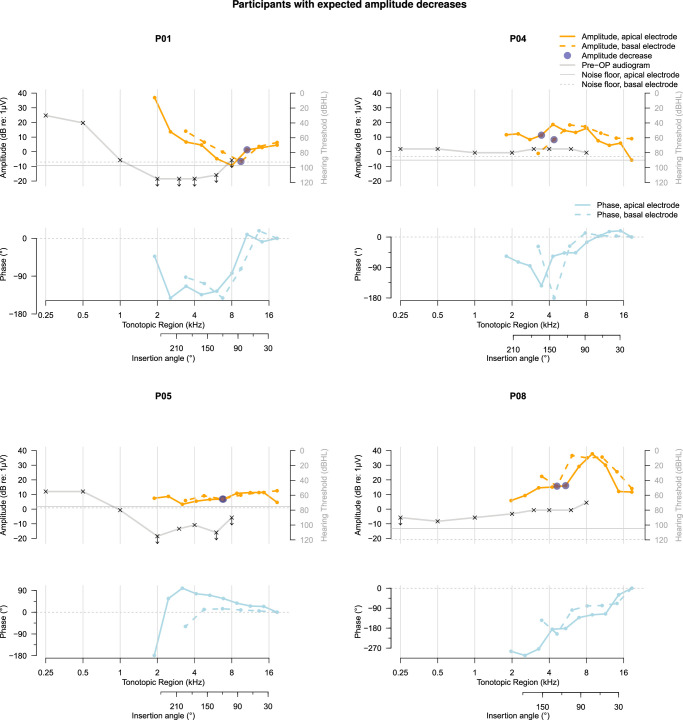
Pure-tone audiogram and ECochG response amplitude and phase for participants in whom an expected amplitude decrease occurred at the basal electrode. Note that the direction of insertion in these figures is from right to left, with the leftmost ECochG response corresponding to complete insertion of the temporary electrode array. Amplitude decreases at the basal electrode contact and the corresponding decreases at the apical electrode contact are marked. In case of multiple sequential amplitude decreases at the basal electrode contact, only the initial drop is indicated. The phase of the ECochG response is shown with respect to the first recording made with each electrode contact and was not corrected for the cycle. In the audiogram, arrows signify that the stimulus was not heard; crosses denote the limits of the audiometer in these cases. dB re: 1 μV indicates decibels relative to 1 microvolt; Pre-OP, preoperative.

**Figure 4:**
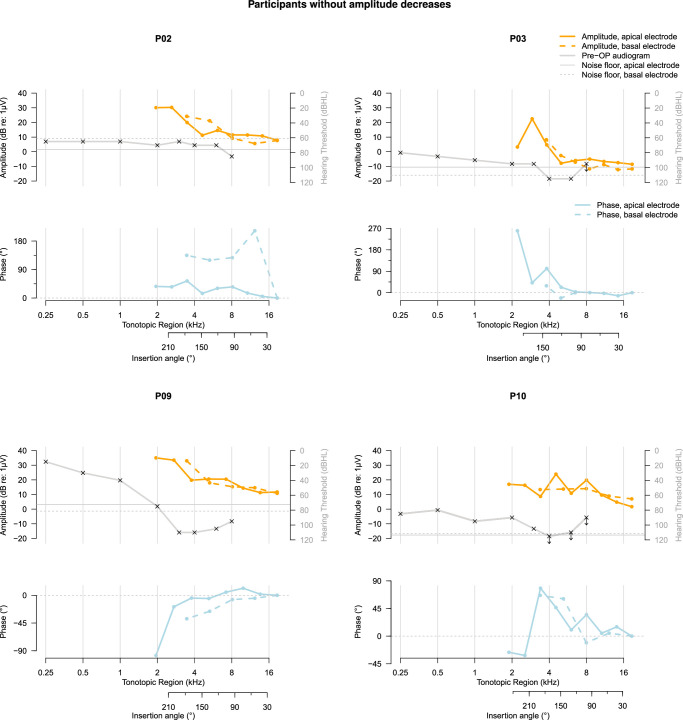
Pure-tone audiogram and ECochG response amplitude and phase for participants in whom no amplitude decreases occurred at the basal electrode. Note that the direction of insertion in these figures is from right to left, with the leftmost ECochG response corresponding to complete insertion of the temporary electrode array. The phase of the ECochG response is shown with respect to the first recording made with each electrode contact and was not corrected for the cycle. In the audiogram, arrows signify that the stimulus was not heard; crosses denote the limits of the audiometer in these cases. dB re: 1 μV indicates decibels relative to 1 microvolt; Pre-OP, preoperative.

**Figure 5:**
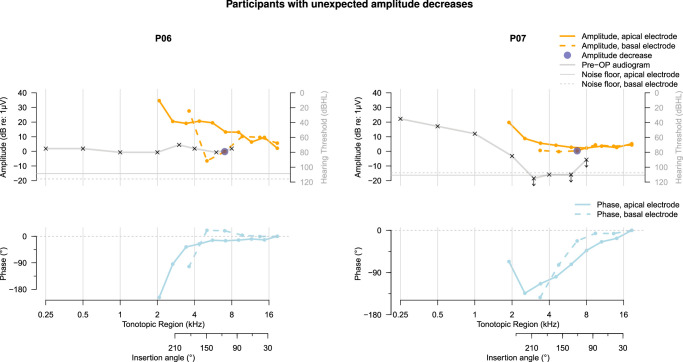
Pure-tone audiogram and ECochG response amplitude and phase for participants in whom an unexpected amplitude decrease occurred at the basal electrode. Note that the direction of insertion in these figures is from right to left, with the leftmost ECochG response corresponding to complete insertion of the temporary electrode array. Decreases in amplitude at the basal electrode contact are marked, noting only the initial drop in case of multiple sequential decreases. The phase of the ECochG response is shown with respect to the first recording made with each electrode contact and was not corrected for the cycle. In the audiogram, arrows signify that the stimulus was not heard; crosses denote the limits of the audiometer in these cases. dB re: 1 μV indicates decibels relative to 1 microvolt; Pre-OP, preoperative.

Overall, recordings of 4 participants (P01, P04, P05, and P08) demonstrated expected, and therefore likely atraumatic amplitude drops at the basal electrode contact, shown in Figure 3. These expected amplitude drops were accompanied by large phase changes in 3 of 4 cases (P01, P04, and P08). Another 4 participants (P02, P03, P09, and P10; Fig. 4) showed no amplitude drops in the basal electrode contact’s recordings, while 2 participants (P06 and P07; Fig. 5) demonstrated unexpected amplitude drops at the basal electrode contact that were not preceded by drops recorded using the apical electrode. These unexpected amplitude drops were not accompanied by simultaneous drops in the apical electrode’s recording, which would be a prerequisite for interpreting them as traumatic.

In the participants with expected amplitude drops at the basal electrode, patterns of ECochG amplitude, and phase change recorded using both electrode contacts resemble each other (Fig. 3). A rough resemblance of ECochG patterns was also observed for the participants showing no amplitude drops recorded using the basal electrode (Fig. 4), except P02 showing a large phase change recorded at the second recording step of only the basal electrode (around 12 mm insertion depth) and P10 showing fluctuations in amplitude at only the apical electrode contact. For the 2 participants with unexpected amplitude drops at the basal electrode contact (Fig. 5), the phases of the ECochG responses recorded using both electrodes resemble each other despite differences between ECochG amplitude patterns.

Although there are some similarities between the participants’ ECochG responses and audiograms, shown in Figures 3–5, their overall relationship does not appears very strong. Participants P01, P07, and P09 show downward sloping audiograms and corresponding basal-to-apical amplitude increases in ECochG responses recorded using the apical electrode. However, P02 and P06 show similar ECochG response amplitude increases with much flatter audiograms, while P05 has a downward sloping audiogram but a mainly decreasing ECochG amplitude toward the apex. Participants P04, P05, and P08, who all showed ECochG amplitude decreases at some point during electrode array insertion, also showed an upward slope in a similar tonotopic region in their audiograms. Note that the upward slope in the higher frequencies of the audiogram, observed slightly between 4 and 6 kHz in some and mainly between 6 and 8 kHz in most participants, is due to the audiometer limit being lower for the highest frequencies.

## Discussion

This study aimed to investigate whether simultaneous ECochG recordings from 2 locations within the cochlea could monitor insertion trauma and help preserve residual acoustic hearing following CI surgery. During stepwise insertions using a short, temporary electrode array, ECochG responses were recorded in 10 of 17 participants. In the remaining 7 cases, it is possible that the electrodes did not advance sufficiently into the cochlea to access the main response generators for low-frequency stimuli, resulting in the absence of recorded responses. This limitation is expected to be less pronounced with longer, standard cochlear implant electrode arrays.

More generally, the recorded potentials are influenced by a complex interplay of hair cell and neural degeneration patterns along the cochlear duct, as well as the spread of electric fields, both of which can vary significantly among individuals.^20^ Findings from a study using an animal model of high-pass noise-induced hearing loss—designed to replicate the high-frequency sloping hearing loss common in CI users—suggest that trauma in the basal cochlear regions can still impact the magnitude of responses to low-frequency tones, even though their response elements are located in far more apical regions.^6^

The response tracks recorded at the apical and more basal contact resemble each other in the majority of cases. In 4 participants (P01, P04, P05, and P08; Fig. 3), response amplitude decreases occur at the same cochlear location, but not at the same time, for both recording electrodes. This temporal offset in amplitude drops suggests that these decreases are not caused by intracochlear trauma, as trauma would be expected to produce a simultaneous amplitude drop—at different recording locations—for both electrodes. Instead, for these cases, the simultaneous ECochG recordings from 2 intracochlear electrodes revealed that the amplitude decreases are atraumatic, unlike standard intracochlear ECochG, which would have suggested trauma based on the recordings. Atraumatic ECochG amplitude decreases may be caused, for example, by destructive interference of response generators, or by reduced hair cell integrity in certain cochlear regions.^7^ They should not be acted upon when using ECochG for providing real-time surgical feedback.

For most participants, there are slight variations between the amplitude and phase patterns along insertion depth recorded by both electrodes, as shown in Figures 3–5. These differences are partly due to inaccuracies in insertion depth estimation and potential trembling or slight movements of the recording electrode, making full resemblance of the graphs infeasible. Robotic insertion with controlled insertion steps may enable more precise comparisons between recording electrodes in the future.^3^

Differences in response amplitudes between electrode contacts may also be a result of differences in electrode impedance. On the one hand, high electrode impedance can record electrical stimulation artifacts because the high impedance acts like an antenna, picking up, and amplifying interferences. Depending on the artifact’s size and its phase relationship with the electrophysiological response, this could either increase or decrease the response amplitude in the recordings. This causes shakiness in the response pattern, as observed in the apical electrode recordings of participant P10. It should be mentioned that electrical artifacts at the acoustic stimulation frequency were absent in the noise floor recording of P10, indicating that this explanation is probably not applicable to these specific recordings. In contrast, high electrode impedance acts as a resistor, diminishing the bioelectrical signals before they reach the amplifier and leading to a decreased response amplitude. Amplitude decreases occurring at only 1 electrode, as in the basal electrode recordings of participants P06 and P07, could be caused by an increase in electrode impedance due to air bubbles on the electrode contact. They could also be caused by changes in the biomechanics or conduction pathways of the scala tympani caused by the electrode array, even in case of atraumatic electrode insertions.

Unfortunately, amplitude decreases resulting from an increase in electrode impedance cannot be clearly distinguished from those caused by trauma, presenting a limitation to the proposed method. In this study, electrode impedance was not controlled because it could not be measured using the Navigator Pro device combined with the ANTS electrode array. However, the noise floors recorded at both electrode contacts were similar within participants, suggesting no large impedance differences were present between contacts at the end of insertion while the noise floors were recorded.

We believe that trauma did not play a role in the unexpected response decreases at the basal electrode contact of P06 and P07 for several reasons. First, previous findings have demonstrated that insertion of the slim, short ANTS electrode array is atraumatic.^13^ Second, for participant P06, the ECochG amplitude recovered again toward insertion completion and acoustic hearing was fully preserved after CI surgery (indicated by a PTA shift of <10 dB 1 mo postimplantation). And third, for both participants, no decrease in ECochG response amplitude was observed at the apical electrode following the detected drop at the basal electrode contact. It is expected that changes in cochlear functionality lead to simultaneous amplitude decreases at both electrode contacts.

Previous studies have demonstrated that phase changes can help distinguish atraumatic from traumatic ECochG response amplitude drops.^7,13–15,18,21^ Specifically, there is evidence that amplitude decreases accompanied by large, near-180-degree phase shifts are indicative of atraumatic events.^14,21^ In this study, 3 of the 4 cases with expected—and therefore likely atraumatic—amplitude drops also exhibited simultaneous large phase shifts, further supporting the findings of previous research.

In previous studies, ECochG signal reductions during insertion have been interpreted as early indicators of possible trauma, and retraction and reinsertion has been proposed as a corrective measure since changes may occur before irreversible damage;^4^ for example, due to contact between the electrode and the basilar membrane. Our study did not include such interventions and was designed as an observational, atraumatic study; therefore, we cannot confirm the effectiveness of this approach in our cohort.

Interpretation of the results was simplified by the use of the ZH-ECochG Bode Plot,^19^ whereby variations in cochlear anatomy were taken into account to enable more precise comparison between audiograms and ECochG responses. The ECochG response patterns mirror the preoperative audiogram in some cases (P01, P07, P09), although they are shifted toward more basal cochlear regions. In other cases (P04, P06, P08), the response patterns and audiogram show no resemblance, making prediction of ECochG responses based on preoperative acoustic hearing thresholds infeasible. Variations in the proportions of intact inner and outer hair cells,^22^ along with different degrees of synaptopathy due to diverse hearing loss etiologies and genetic profiles,^23^ can explain the lack of relationship between ECochG responses and the audiogram.^20^

Limitations to this study include the relatively small number of participants and the unknown cause of the unexpected amplitude drop observed in 2 cases, which may have resulted from an uncertainty in the recording setup. However, while there is an extensive body of research on ECochG for monitoring residual hearing during CI surgery, this study is the first to propose and investigate simultaneous recordings from 2 intracochlear locations. This novel approach may overcome limitations to standard intracochlear ECochG with a single electrode by distinguishing traumatic and atraumatic amplitude drops. Looking ahead, CI manufacturers should enable simultaneous recordings from 2 electrodes in standard CI surgeries to further validate this method and explore its clinical applicability.

## Conclusion

n this study, the amplitude and phase of ECochG responses recorded using 2 intracochlear electrode contacts showed a large overall resemblance, with changes in both recordings occurring nonsimultaneously but at the same location within the cochlea. ECochG amplitude decreases occur regularly during insertion of an electrode array into the cochlea and do not necessarily indicate trauma. Trauma would be expected to produce a simultaneous amplitude drop—at different recording locations—for both electrodes. In contrast, a drop at the basal electrode that is preceded by a drop in the ECochG recording at the apical electrode, with both drops occurring nonsimultaneously but approximately at the same location, is likely atraumatic. These atraumatic drops may also be associated with large phase shifts. Overall, our results suggest that intracochlear ECochG using 2 recording electrodes is a promising method to monitor inner ear function during CI surgery.

## References

[1] Campbell L Kaicer A Sly D . Intraoperative real-time cochlear response telemetry predicts hearing preservation in cochlear implantation. Otol Neurotol 2016;37:332–8. 26859542 10.1097/MAO.0000000000000972

[2] Harris MS Riggs WJ Koka K . Real-time intracochlear electrocochleography obtained directly through a cochlear implant. Otol Neurotol 2017;38:e107–13. 28498269 10.1097/MAO.0000000000001425

[3] Scheperle R Etler C Oleson J . Evaluation of real-time Intracochlear Electrocochleography for guiding Cochlear implant electrode Array position. J Clin Med 2023;12:7409. 38068461 10.3390/jcm12237409 PMC10707171

[4] Bester C Collins A Razmovski T . Electrocochleography triggered intervention successfully preserves residual hearing during cochlear implantation: results of a randomised clinical trial. Hear Res 2022;426:108353. 34600798 10.1016/j.heares.2021.108353

[5] Kashani RG Kocharyan A Bennion DM . Combining intraoperative electrocochleography with robotics-assisted electrode array insertion. Otol Neurotol 2024;45:143–9. 38206061 10.1097/MAO.0000000000004094 PMC10786337

[6] Choudhury B Adunka OF DeMason CE Ahmad FI Buchman CA Fitzpatrick DC . Detection of intracochlear damage with cochlear implantation in a gerbil model of hearing loss. Otol Neurotol 2011;32:1370–8. 21921858 10.1097/MAO.0b013e31822f09f2 PMC3338854

[7] Giardina CK Brown KD Adunka OF . Intracochlear electrocochleography: Response patterns during cochlear implantation and hearing preservation. Ear Hear 2019;40:833. 30335669 10.1097/AUD.0000000000000659 PMC6534483

[8] Soulby A Connor S Jiang D Nunn T Boyle P Pai I . Establishing reproducibility and correlation of cochlear microphonic amplitude to implant electrode position using intraoperative electrocochleography and postoperative cone beam computed tomography. Ear Hear 2021;42:1263. 33813521 10.1097/AUD.0000000000001010 PMC8378545

[9] Kiefer J Böhnke F Adunka O Arnold W . Representation of acoustic signals in the human cochlea in presence of a cochlear implant electrode. Hear Res 2006;221:36–43. 16962268 10.1016/j.heares.2006.07.013

[10] Bester C Dalbert A Collins A Razmovski T Gerard JM O’Leary S . Electrocochleographic patterns predicting increased impedances and hearing loss after cochlear implantation. Ear Hear 2023;44:710–20. 36550618 10.1097/AUD.0000000000001319

[11] Walia A Shew MA Lefler SM . Is characteristic frequency limiting real-time electrocochleography during cochlear implantation? Front Neurosci 2022;16:915302. 35937872 10.3389/fnins.2022.915302 PMC9354607

[12] Saoji AA Graham MK Adkins WJ . Multi-frequency electrocochleography and electrode scan to identify electrode insertion trauma during cochlear implantation. Brain Sci 2023;13:330. 36831873 10.3390/brainsci13020330 PMC9954676

[13] Dalbert A Sijgers L Grosse J . Simultaneous intra-and extracochlear electrocochleography during electrode insertion. Ear Hear 2021;42:414–24. 32826509 10.1097/AUD.0000000000000935

[14] Sijgers L Pfiffner F Grosse J . Simultaneous intra-and extracochlear electrocochleography during cochlear implantation to enhance response interpretation. Trends Hear 2021;25:2331216521990594. 33710919 10.1177/2331216521990594 PMC7958165

[15] Weder S Bester C Collins A Shaul C Briggs RJ O’Leary S . Real time monitoring during cochlear implantation: increasing the accuracy of predicting residual hearing outcomes. Otol Neurotol 2021;42:e1030–6. 33859138 10.1097/MAO.0000000000003177

[16] Sijgers L Sorensen T Soulby A . Classification of acoustic hearing preservation after cochlear implantation using electrocochleography. Trends Hear 2023;27:23312165231220996. 10.1177/23312165231220997 PMC10729624 38105510

[17] Skarżyński PH Lorens A Walkowiak A Polak M Skarżyński H . Multi-frequency intraoperative monitoring of hearing preservation during cochlear implantation. Life 2022;12:636. 35629304 10.3390/life12050636 PMC9143534

[18] Koka K Riggs WJ Dwyer R . Intra-cochlear electrocochleography during cochear implant electrode insertion is predictive of final scalar location. Otol Neurotol 2018;39:e654–9. 30113557 10.1097/MAO.0000000000001906 PMC6097527

[19] Geys M Sijgers L Dobrev I . ZH-ECochG bode plot: a novel approach to visualize electrocochleographic data in cochlear implant users. J Clin Med 2024;13:3470. 38929998 10.3390/jcm13123470 PMC11205027

[20] van Gendt MJ Koka K Kalkman RK . Simulating intracochlear electrocochleography with a combined model of acoustic hearing and electric current spread in the cochlea. J Acoust Soc Am 2020;147:2049–60. 32237816 10.1121/10.0000948

[21] Buechner A Bardt M Haumann S Geissler G Salcher R Lenarz T . Clinical experiences with intraoperative electrocochleography in cochlear implant recipients and its potential to reduce insertion trauma and improve postoperative hearing preservation. PLoS One 2022;17:e0266077. 35452461 10.1371/journal.pone.0266077 PMC9032378

[22] Ryan A Dallos P . Effect of absence of cochlear outer hair cells on behavioural auditory threshold. Nature 1975;253:44–6. 1110747 10.1038/253044a0

[23] Angeli S Lin X Liu XZ . Genetics of hearing and deafness. Anat Rec 2012;295:1812–29. 10.1002/ar.22579 PMC4523052 23044516

